# Hepatocellular carcinoma with extrahepatic blood supply from right renal artery

**DOI:** 10.1093/jscr/rjab391

**Published:** 2021-10-11

**Authors:** Amir Humza Sohail, Ahmad Musa, Muhammad Salman Khan, Hassan Raza Hashmi, Basit Salam, Collin E M Brathwaite

**Affiliations:** NYU Langone Hospital – Long Island, Mineola, NY, USA; NYU Langone Hospital – Long Island, Mineola, NY, USA; University of Tennessee Health Science Center Bookstore, Radiology, Memphis, TN, USA; NYU Langone Hospital – Long Island, Mineola, NY, USA; The Aga Khan University, Radiology, Karachi, Sindh, Pakistan; NYU Langone Hospital – Long Island, Mineola, NY, USA

## Abstract

Extrahepatic blood supply is seen in around 17–27% of hepatocellular carcinoma lesions. Evidence suggests that this extrahepatic supply most commonly originates from a right intercostal artery (70–83%) followed by left intercostal, omental and right renal arteries. Thus a comprehensive knowledge of variations in standard vascular anatomy and cognisance of factors influencing or predicting extrahepatic blood supply in HCC is instrumental in ensuring the success of surgical and interventional procedures. We present the unusual case of a 66-year-old male with HCC in Segment I of the liver with aberrant blood supply from the right renal artery in the absence of any risk factors for extrahepatic circulation. He successfully underwent transarterial chemoembolization. There was no evidence of residual disease on repeat imaging.

## INTRODUCTION

Extrahepatic blood supply is seen in around 17–27% of hepatocellular carcinoma lesions. Evidence suggests that this extrahepatic supply most commonly originates from a right intercostal artery (70–83%) followed by left intercostal, omental and right renal arteries. Thus a comprehensive knowledge of variations in standard vascular anatomy and cognisance of factors influencing or predicting extrahepatic blood supply in hepatocellular carcinoma (HCC) is instrumental in ensuring the success of surgical and interventional procedures. Transcatheter arterial chemoembolization is an endovascular procedure that attempts to restrict blood supply to the tumor and is the standard locoregional treatment for HCC in patients that fail to qualify for ablation or surgical resection. The success of trans-arterial chemo-embolization (TACE) hinges upon the ability to chemoembolize all feeding vessels including extrahepatic feeders. Herein, we present the unusual case of a 66-year-old male with HCC with aberrant blood supply from the right renal artery in the absence of any risk factors for extrahepatic circulation and successfully underwent TACE.

## CASE DESCRIPTION

**Figure 1:**
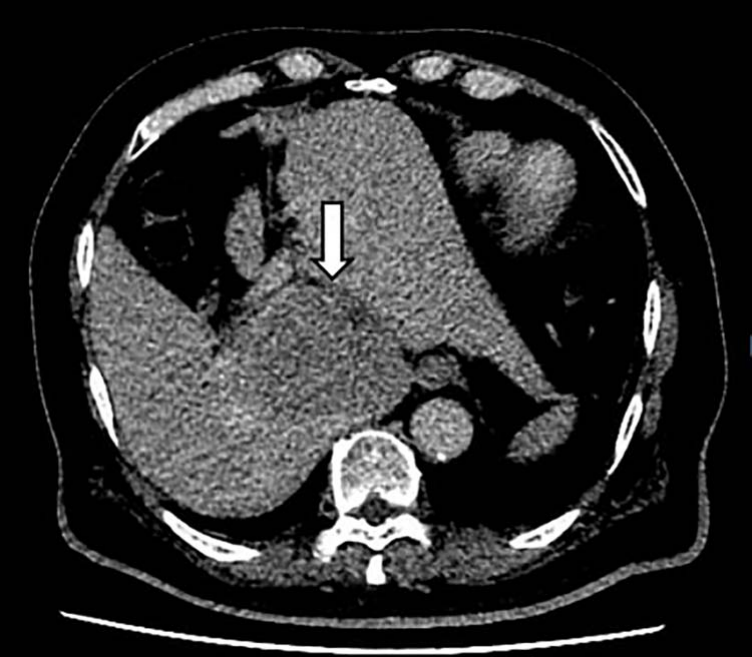
CT scan axial section delayed phase showing a 3.2 × 3.0-cm lesion (arrow) with early washout in Segment I of liver, consistent with hepatocellular carcinoma.

**Figure 2:**
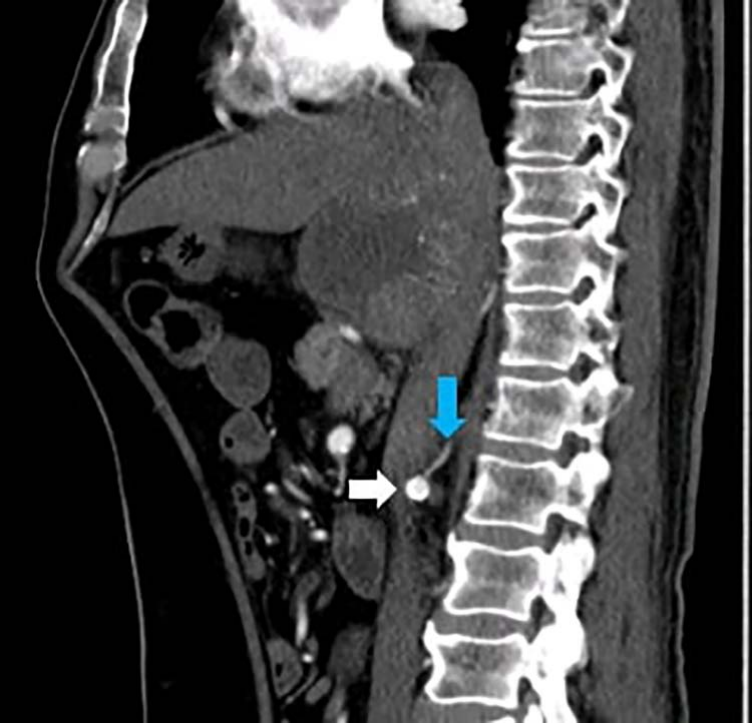
CT scan sagittal section arterial phase showing a branch (blue arrow) originating from the right renal artery (white arrow) supplying the lesion in Segment I of the liver.

**Figure 3:**
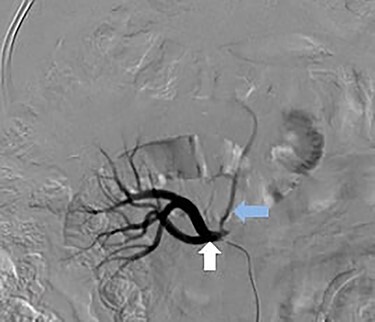
Selective catheter angiogram of right renal artery (white arrow) showing a branch near its origin (blue arrow) supplying the hepatic lesion.

**Figure 4:**
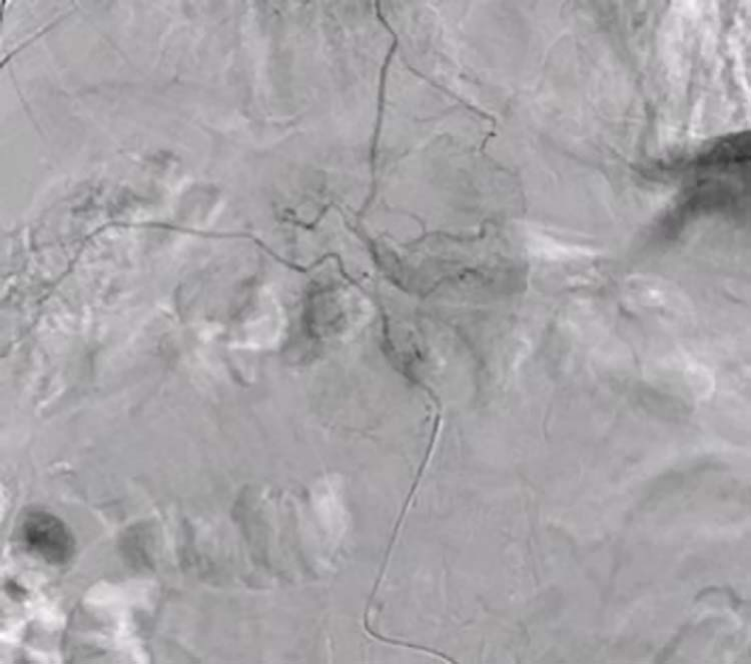
Superselective delayed phase angiogram of right renal artery using microcatheter showing well-defined tumor blush.

A 66-year-old male was referred to our institution for management of a liver lesion found incidentally on abdominal imaging. There was no significant past medical or surgical history, and he was asymptomatic. On physical examination, the patient was vitally stable and all systemic examinations were unremarkable. Contrast-enhanced triple phase abdominal computed tomography (CT) scan showed an arterial enhancing lesion in Segment I of liver with early washout, consistent with HCC (Fig. 1). This lesion received blood supply from the right renal artery (Fig. 2). Ultrasound-guided biopsy was performed to confirm the diagnosis of HCC, and subsequently the patient successfully underwent TACE. Figures 3 and 4 show the angiographic findings of the right renal artery branch supplying the lesion. Repeat CT scan at 6-week follow-up showed no evidence of residual disease.

## DISCUSSION

The prevalence of extrahepatic circulation in HCC is well-documented [1]. Initial studies on extrahepatic circulation in patients with HCC pointed towards the possibility of ligation of hepatic artery, a now obsolete treatment for HCC, as the causative factor [2]. Recent evidence suggests that a previous history of TACE procedures is an important predisposing factor for extrahepatic circulation in HCC [1]. However, in patients without a previous history of intervention, several factors can predict the development extrahepatic circulation. Tumor size >5 cm, peripheral location of HCC—especially tumors in close proximity of the diaphragm, hepatic ligaments or the bare area of liver i.e. Segments VII and VIII—owing to their often direct contact with extrahepatic structures, exophytic tumors with omental or peritoneal adhesions and extrahepatic tumor infiltration often major predictors of extrahepatic circulation [3, 4]. Further, prior abdominal surgery or a history of ruptured HCC are other important risk factors for development of extrahepatic circulation [5–7]. It is noteworthy that our patient did not have any of the above-mentioned risk factors. Importantly, the HCC lesion in our patient, located in Segment I, was only 3.0 cm × 3.2 cm in size.

In HCC, extrahepatic blood supply from right renal artery occurs in 3.5–12.0% of cases [3, 8]. Most commonly lesions in Segment VI, followed by Segment VIII tumors, develop extrahepatic supply from the superior capsular branch of right renal artery [9]. Origin of feeding vessel(s) directly from right renal artery, especially near its origin, as seen in our case, is extremely rare. Notably, preprocedure CT imaging generally has poor sensitivity for detection of extrahepatic circulation from the right renal artery. Evidence on the success rate for TACE via right renal artery feeders is variable, with the success rate ranging from 41 to 100%, and no major complications reported in literature [3].

## LEARNING POINTS

- Hepatocellular carcinoma lesions may rarely have blood supply directly from the right renal artery.
- Angiography can help to confirm diagnosis of such lesions with isolated blood supply from the right renal artery.
- In such lesions, transarterial chemoembolization should be considered and can result in complete resolution of the lesion.
