# Erratum to: Hepatocellular carcinoma with extrahepatic blood supply from right renal artery

**DOI:** 10.1093/jscr/rjab511

**Published:** 2021-11-03

**Authors:** Amir Humza Sohail, Ahmad Musa, Muhammad Salman Khan, Hassan Raza Hashmi, Basit Salam, Collin E M Brathwaite

**Affiliations:** NYU Langone Hospital – Long Island, Mineola, NY, USA; NYU Langone Hospital – Long Island, Mineola, NY, USA; University of Tennessee Health Science Center Bookstore, Radiology, Memphis, TN, USA; NYU Langone Hospital – Long Island, Mineola, NY, USA; The Aga Khan University, Radiology, Karachi, Sindh, Pakistan; NYU Langone Hospital – Long Island, Mineola, NY, USA

In the originally published version of this manuscript, there were errors in the list of authors. It should read “Amir Humza Sohail, Ahmad Musa, Muhammad Salman Khan, Hassan Raza Hashmi, Basit Salam and Collin E.M. Brathwaite” instead of “Sohail Amir, Ahmad Musa, Khan Muhammad Salman, Hassan Hashmi, Salam Basit and Collin E.M. Brathwaite”. This error has now been corrected online.

